# Heavy atom effects in the Paternò–Büchi reaction of pyrimidine derivatives with 4,4’-disubstituted benzophenones

**DOI:** 10.3762/bjoc.7.16

**Published:** 2011-01-26

**Authors:** Feng-Feng Kong, Jian-Bo Wang, Qin-Hua Song

**Affiliations:** 1 Department of Chemistry, University of Science and Technology of China, Hefei 230026, Anhui, P. R. China

**Keywords:** benzophenone, heavy atom effect, Paternò–Büchi reaction, regioselectivity, triplet diradical

## Abstract

The regioselectivity and the photochemical efficiency were investigated in the Paternò–Büchi reaction of 1,3-dimethylthymine (DMT) and 1,3-dimethyluracil (DMU) with benzophenone (**1b**) and some 4,4’-disubstituted derivatives (dimethoxy (**1a**), difluoro (**1c**), dichloro (**1d**), dibromo (**1e**) and dicyano benzophenone (**1f**)) that gives rise to two regioisomeric oxetanes, **2** and **3**. The regioselectivity (the ratio of **2**/**3**) decreased gradually for both DMT/DMU photochemical systems from **1a** to **1f**. That is, a halogen atom as an electron-withdrawing group (EWG) has a pronounced effect on the regioselectivity. However, the photochemical efficiency of the **1e** systems did not show the expected increase, but decreased relative to systems with **1b.** Temperature effects on the regioselectivity of **1b–e** systems showed some interesting features for systems with heavy atoms (including the **1d** and **1e** systems), such as higher inversion temperatures, and an entropy-controlled regioselectively whereas the regioselectivity for two other systems (**1b** and **1c**) is enthalpy–entropy controlled. A heavy atom effect is suggested to be responsible for these unusual phenomena based on the triplet-diradical mechanism of the Paternò–Büchi reaction.

## Introduction

The regio- and stereoselectivity in the Paternò–Büchi reaction, which is a photochemical [2 + 2] cycloaddition of a carbonyl compound with an olefin, has been extensively studied [1–4]. The ene–carbonyl photocycloaddition generally proceeds through attack of the excited carbonyl state (singlet or triplet or both) on a ground-state olefin. For aromatic carbonyl compounds, the reaction is a triplet cycloaddition, that is, a triplet-excited carbonyl compound adding to an olefin to yield a triplet 1,4-diradical intermediate, which undergoes intersystem crossing (ISC) to produce a singlet 1,4-diradical. Ring-close of the latter gives an oxetane. The higher selectivity observed for the triplet reaction is rationalized by the optional conformation of the intermediate 2-oxabutane-1,4-diyl for ISC to the singlet diradical, which is preferentially controlled by spin-orbit coupling, thus leading to substantial control of regio- and stereoselectivity [5–13].

The “heavy atom effect” is a term which has been used to describe the influence of “heavy atom” substitution on a spin-forbidden transition such as various intersystem crossings. If heavy atoms are present in a Paternò–Büchi reaction, spin-transition processes would be affected, and this may lead to interesting results.

Abe et al. [14] investigated the effect of a sulfur atom on the stereoselective formation of oxetanes in Paternò–Büchi reaction of aromatic aldehydes with silyl *O*,*S*-ketene acetals to give *trans*-3-siloxyoxetanes and found that this was ca. 70/30 to 90/10. The trans-selectivity is explained by the sulfur atom effect in the silyl *O*,*S*-ketene acetal which controls the approach direction of the electrophilic oxygen of triplet n,π* aldehyde to the nucleophilic alkene. A fast ISC process of the triplet 1-alkylthio-1-siloxy-2-oxatetramethylene 1,4-diradical in competition with the bond rotation was proposed [14]. Griesbeck et al. [15] observed substantial ^2^H-magnetic isotope effects on the diastereoselectivity of triplet photocycloaddition reactions. Weaker isotope effects on the diastereoselectivity were detected for the reaction of α-deuterated propionaldehyde [15].

**Scheme 1:**
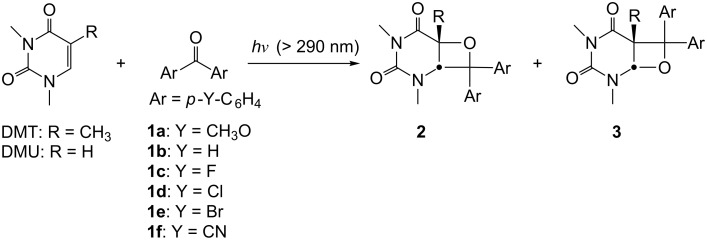
The Paternò–Büchi reaction of DMT/DMU with benzophenones to generate two regioisomeric photoproducts.

In this work, we have investigated the Paternò–Büchi reaction of 1,3-dimethylthymine (DMT) and 1,3-dimethyluracil (DMU) with benzophenone (**1b**) and its 4,4’-disubstituted derivatives **1a** and **1c**–**1f** with the formation of the regioisomeric oxetanes **2** and **3** (Scheme 1). By changing the halogen at para positions in the benzophenones, the photochemical efficiency and the regioselectivity were significantly affected, and the effects cannot be considered as a pure electronic effect (of the electron-withdrawing groups, EWGs), by comparing the observations with those of systems of **1a** (with electron-donating groups, EDGs), and **1b** and **1f** (also with EWGs). However, as a heavy atom effect, observations above can be rationalized based on the triplet mechanism of the Paternò–Büchi reaction.

## Results and Discussion

### Substituent effects

To investigate substituent effects of benzophenones in the Paternò–Büchi reaction, photochemical reactions of DMT/DMU with **1a**–**f** in acetonitrile-*d*_3_ were performed in Pyrex NMR tubes. The regioselectivity (the ratio of **2**/**3**) and the yield were measured directly from the ^1^H NMR spectra of crude product mixtures and are listed in Table 1. The substituent effect of benzophenones on the regioselectivity (**2**/**3**) is similar to our previous observations [10], a gradual decrease according to their electronic effect from **1a** to **1e**.

**Table 1:** Substituents (Y) of benzophenones dependence on the regioselectivity (**2**/**3**) and the efficiency in the Paternò–Büchi reactions of DMT/DMU with 4,4’-disubstituted benzophenones **1a**–**1f**^a^.

	Y	DMT^b^		DMU^c^	
		Yield%	**2/3**	Yield%	**2/3**

**1a**	CH_3_O	25	52:48	19	> 95:5^d^
**1b**	H	52	55:45	51	64:36
**1c**	F	53	56:44	64	63:37
**1d**	Cl	77	38:62	70	56:44
**1e**	Br	46	33:67	28	53:47
**1f**	CN	82	14:86	75	39:61

^a^Average of two parallel determinations, DMT (DMU)/benzophenones = 10 mM:10 mM, in *d*_3_-acetonitrile, irradiation at 10 °C with 125 W high-pressure Hg lamp, values determined by ^1^H NMR of the crude product mixture, the experimental error < 5%. ^b^Irradiation for 30 min. ^c^Irradiation for 90 min. ^d^Oxetane **3a_2_** was not detected, see Experimental section.

In our previous papers [10,13], the photochemical [2 + 2] cycloadditions of DMT and DMU with benzophenones generate two series of regioisomeric oxetanes, **2** and **3**, via 1,4-diradical intermediates, and reveal notable substituent effects on the regioselectivity and the photochemical efficiency. The reactions initiated by triplet benzophenones with EDGs give a higher proportion of **2** and a lower photochemical efficiency, whilst benzophenones with EWGs yield a lower proportion of **2** and have a higher efficiency.

The data in Table 1 show that the regioselectivity (**2**/**3**) and the photochemical efficiency correlates clearly with electronic property of substituents. The benzophenones with EWGs give more efficient Paternò–Büchi reactions (except **1e** systems) and lower ratios of **2**/**3**, and the benzophenones with EDGs undergo less efficient Paternò–Büchi reactions and have higher ratios of **2**/**3**, in accord with our previous observations [10,13]. However, the photochemical efficiency of the **1e** systems decreased significantly. According to our understanding of these oxetanes [10], this low efficiency was considered to be due to poor stability of the photoproducts, in particular oxetanes **3**.

**Figure 1:**
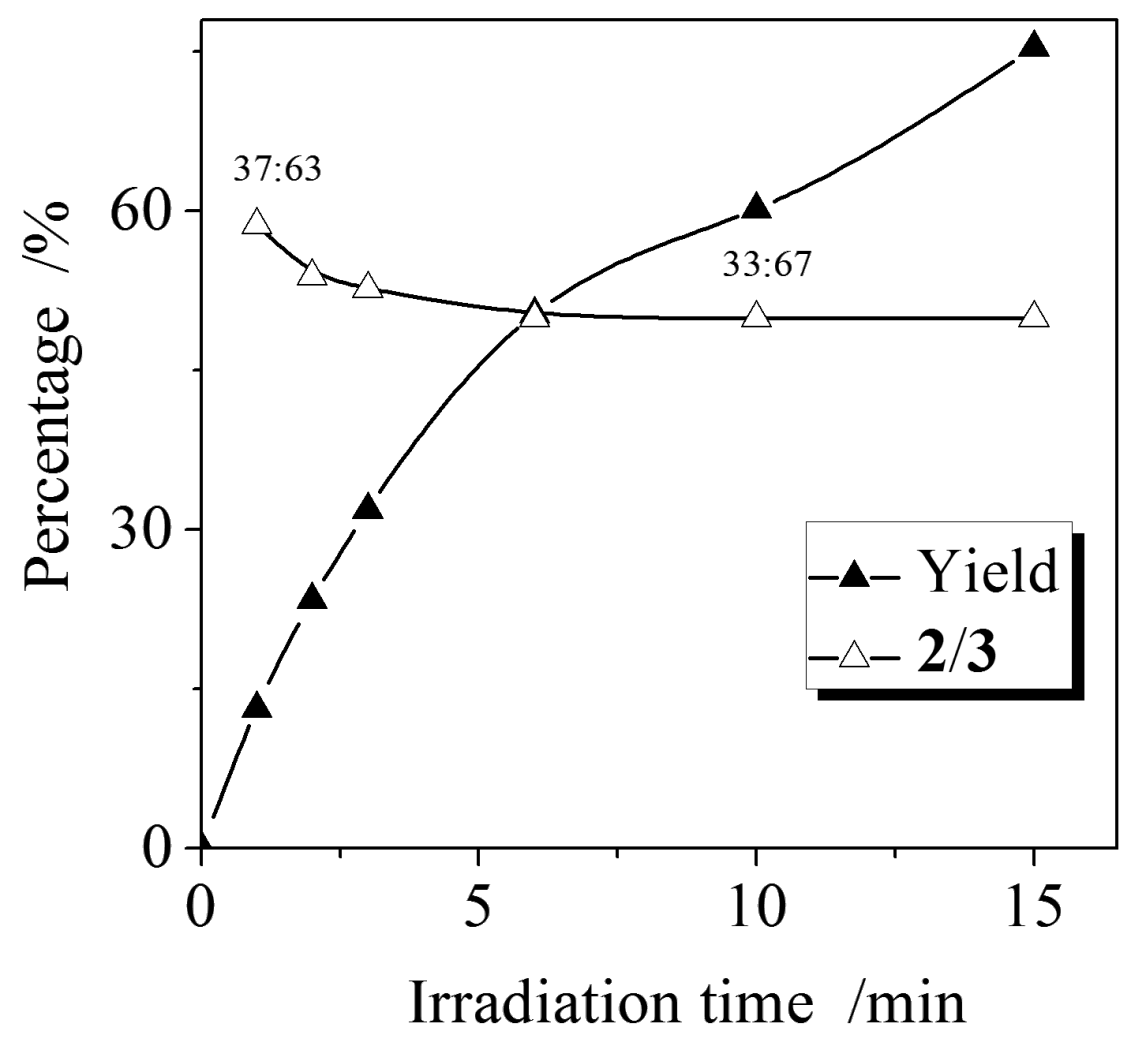
The yield and the ratio of **2**/**3** at different reaction times in the Paternò–Büchi reaction of DMT with **1e** (DMT:**1e** = 5 mM:5 mM, irradiation at 10 °C).

To verify this speculation, the yield and the regioselectivity were tracked over an irradiation time of 15 min for the DMT-**1e** (5 mM/5 mM) system (Figure 1). Figure 1 clearly shows that the yield increases with irradiation time and the ratio of **2**/**3** is slightly higher (37:63) during the initial irradiation period (1–3 min), and then becomes constant (33:67) on further irradiation (> 3 min). However, this change is within the experimental error of 5%. According to our understanding of the stability of oxetanes, **3** are less stable than **2**. The constant ratio of **2**/**3** implies that no significant decomposition of the photoproduct oxetanes occurs in the photochemical reaction. In other words, the stability of photoproducts in the systems is not responsible for the low yields. Therefore, the effect of halo-substituted benzophenones on the Paternò–Büchi reaction is not a “pure” substituent effect.

### Temperature effects

In our previous papers [9,11], the photochemical [2 + 2] cycloadditions of DMT/DMU with benzophenones revealed notable temperature effects on the regioselectivity and the photochemical efficiency. We have demonstrated that the temperature-dependent regioselectivity is derived from the conformational properties of the intermediate triplet 1,4-diradicals. The observations show that the reaction temperature influences the regioselectivity by changing the populations of two regioisomeric diradicals as a result of differences in the potential energies of two stable conformers, the productive conformation of the triplet diradical and the unproductive conformation of the triplet diradical, for each regioisomeric diradical [9,11].

To investigate further the temperature effects in four systems with 4,4’-dihalo-substituted benzophenones, we carried out the Paternò–Büchi reactions of DMT with **1b–1e** over a temperature range of −30 to 70 °C: Notable temperature effects were observed. Both the photochemical efficiency and the regioselectivity (**2**/**3**) decreased with increasing temperature from the general trend (Table 2).

**Table 2:** Temperature dependence on the regioselectivity (**2**/**3**) and the yields in the Paternò–Büchi reactions of DMT with compounds **1b**–**e**^a^.

Temp./°C	**2/3** (yield %)			
	**1b**	**1c**	**1d**	**1e**

−27.4	70:30 (63.9)	73:27 (62.2)	54:46 (75.6)	50:50 (37.6)
−21.4	68:32 (61.1)	70:30 (58.4)	52:48 (68.5)	46:54 (37.5)
−11.5	64:36 (62.5)	67:33 (62.3)	46:54 (69.6)	40:50 (44.4)
−0.9	61:39 (51.0)	62:38 (52.5)	42:58 (74.7)	36:64 (38.3)
9.9	56:44 (46.9)	58:42 (49.7)	39:61 (75.0)	31:69 (38.5)
20.1	52:48 (44.0)	56:44 (47.4)	34:66 (74.4)	30:70 (32.8)
30.0	48:52 (43.1)	51:49 (45.9)	30:70 (63.4)	25:75 (29.2)
40.0	41:59 (36.3)	41:59 (43.0)	26:74 (67.4)	23:77 (26.7)
49.5	37:63 (32.2)	35:65 (43.8)	21:79 (62.8)	18:82 (22.4)
60.0	31:69 (24.1)	29:71 (43.5)	17:83 (63.5)	14:86 (14.5)
69.1	27:73 (25.8)	25:75 (41.3)	14:86 (59.8)	10:90 (11.4)

^a^See Table 1.

Table 2 shows clearly that efficiencies of the **1b** system are lower than those of the **1c** and **1d** systems except for the values at the initial three temperatures, i.e., the efficiency of **1b** system is the most sensitive to reaction temperature, and the efficiency of **1e** system is the lowest among the four systems studied. In addition, the data show that a higher reaction temperature is unfavorable for Paternò–Büchi reactions.

**Figure 2:**
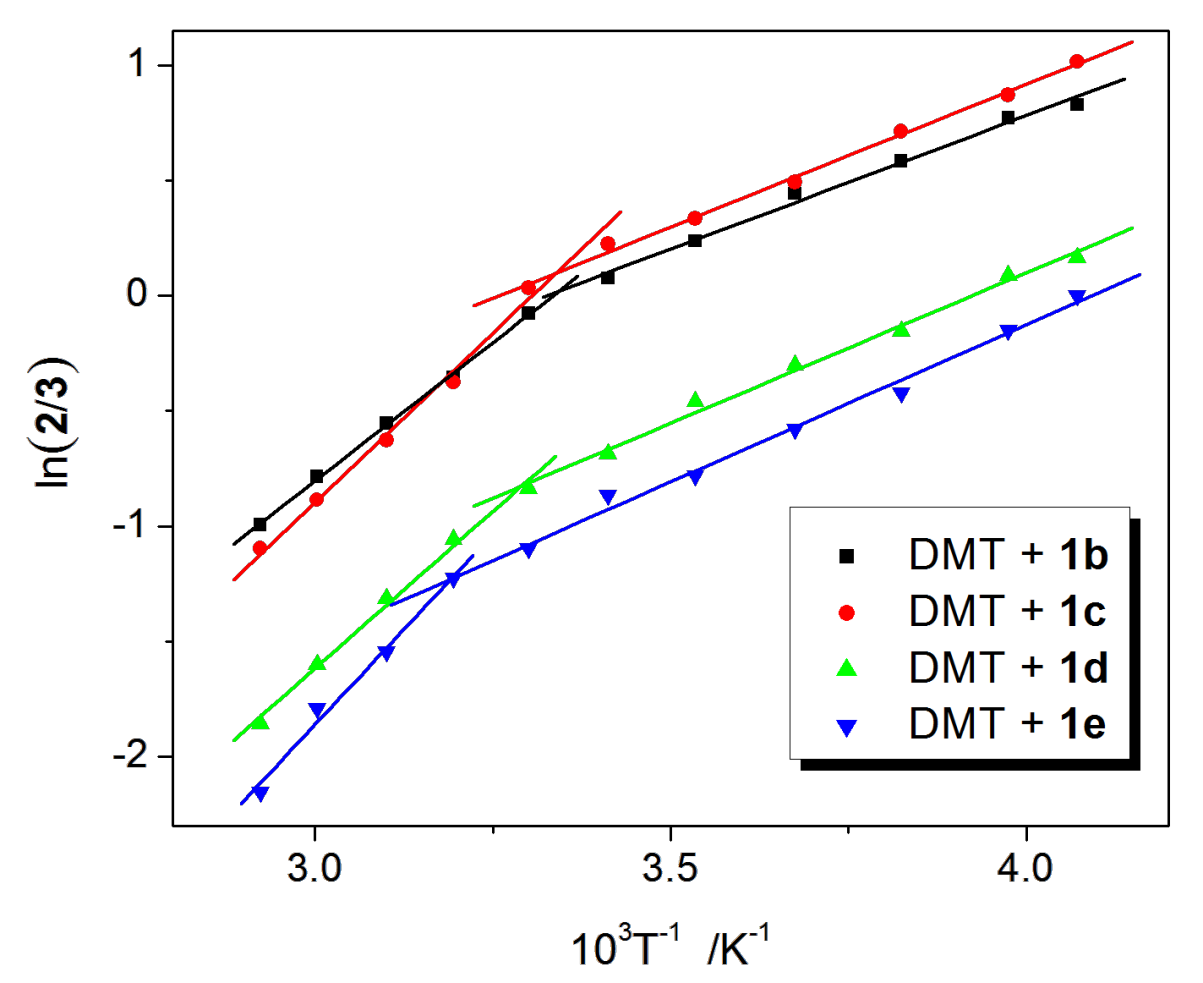
Eyring plots for the photoreaction of DMT with compounds **1b**–**e**.

The regioselectivity (**2**/**3**) data in Table 2 have been plotted in Figure 2 against the inverse absolute temperature according to the Eyring formalism [16].

[1]
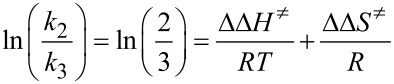


Where *k*_2_ and *k*_3_ are the overall rate constants of the reactions leading to the two regioisomers **2** and **3**, respectively.

The Eyring plots obtained are nonlinear across the whole temperature range. The nonlinear Eyring plot is indicative of a change of the selectivity-determining step during the change in the reaction temperature [1,17–19].

Although the Eyring plots obtained were nonlinear over whole temperature range, strict linearities (correlation coefficients R > 0.99) on both sides of inversion points were found. The temperature at the point of inversion is called the inversion temperature, *T*_inv_, of the system. The temperatures increase gradually from H- to Br-substituted Paternò–Büchi systems (Table 2). In our previous paper [11], the temperature effect of the Paternò–Büchi reaction DMU with three benzophenones, **1b**, **1c** and **1f**, was investigated, and similar inversion temperatures could be obtained from the Eyring plots, 295 K for **1b**, 294 K for **1c** and 291 K for **1f**. Although CN is a strong EWG, **1f**-DMU system did not give a high inversion temperature. Hence, this result indicates that the halogen (Cl or Br) acts not as a pure EWG but as a heavy atom and induces a higher inversion temperature.

According to the Eyring theory, when this relationship is plotted, the slope corresponds to the difference in the overall activation enthalpies (ΔΔ*H*^≠^) and the intercept represents the difference in the overall activation entropies (ΔΔ*S*^≠^) (Figure 2). The inversion temperature reveals two sets of activation parameters (ΔΔ*H*_1_^≠^ and ΔΔ*S*_1_^≠^ (*T* > *T*_inv_), ΔΔ*H*_2_^≠^ and ΔΔ*S*_2_^≠^ (*T* < *T*_inv_)), which were obtained from the slope and the intercept of the linear plot for each system. Table 3 presents the parameters of activation ΔΔ*H*_1,2_^≠^ and ΔΔ*S*_1,2_^≠^ values. These large parameters of activation are unprecedented, ΔΔ*H*_1_^≠^ values range from −19.9 to −27.5 kJ mol^−1^ and are much higher than the published values −4.2 kJ mol^−1^ [1], 4.3 kJ mol^−1^ [8] and −4.8 kJ mol^−1^ [7]. Therefore, the regioselectivity in the Paternò–Büchi reaction is strongly temperature-dependent. Moreover, these activation parameters (ΔΔ*H*^≠^ and ΔΔ*S*^≠^) increase gradually from the F- to Br- benzophenones systems, with the exception of ΔΔ*H*_1_^≠^ for **1d**.

**Table 3:** Inversion temperature, *T*_inv_ and parameters of activation, ΔΔ*H*_1_^≠^, ΔΔ*S*_1_^≠^ (*T* > *T*_inv_), and ΔΔ*H*_2_^≠^, ΔΔ*S*_2_^≠^ (*T* < *T*_inv_).

	ΔΔ*H*_1_^≠^ ΔΔ*H*_2_^≠^ /kJ mol^−1^	δΔΔ*H*^≠^ /kJ mol^−1^	ΔΔ*S*_1_^≠^ ΔΔ*S*_2_^≠^ /J mol^−1^K^−1^	δΔΔ*S*^≠^ /J mol^−1^K^−1^	*T*_inv_ /K

**1b**	−19.9 −9.6	10.9	−66.4 −32.0	34.4	299.0
**1c**	−24.4 −10.3	14.1	−80.7 −33.5	47.2	299.3
**1d**	−22.6 −10.8	11.8	−81.3 −42.5	38.8	303.6
**1e**	−27.5 −11.4	16.1	−97.9 −46.4	51.5	313.6

In addition, the values of ΔΔ*H*^≠^ are similar to *T*_inv_ΔΔ*S*^≠^ for the **1b** and **1c** systems since the ratio of **2**/**3** is ~50:50. However, the values of ΔΔ*H*^≠^ are less than *T*_inv_ΔΔ*S*^≠^ for two other systems (−2.1 J/mol for **1d**, −3.2 kJ/mol for **1e**). In other words, this is an entropy-determined selection for the regioselectivity over the whole temperature range investigated.

### Interception of heavy atom effects

**Scheme 2:**
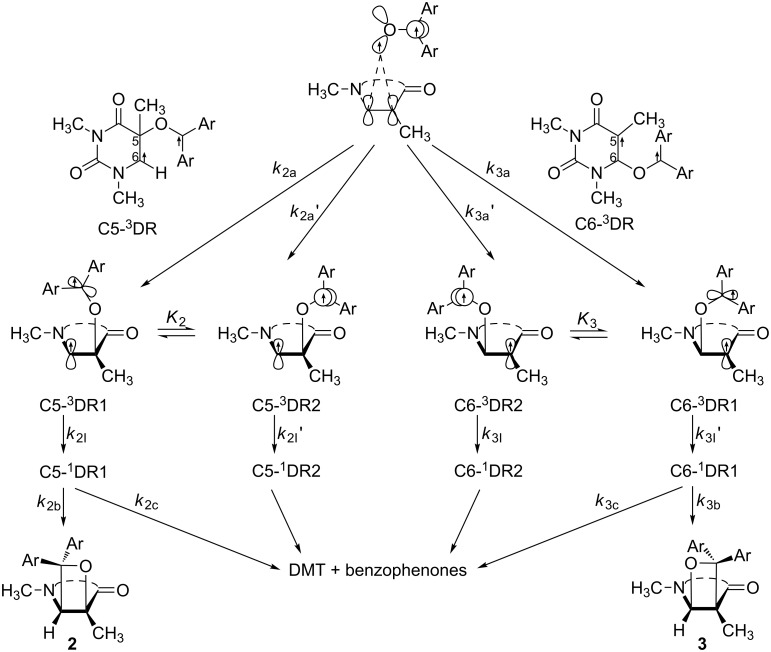
The formational processes of two regioisomers in the Paternò–Büchi reaction of DMT/DMU with benzophenones [9].

Based on the triplet mechanism of the Paternò–Büchi reaction, it is possible to have a more detailed discussion on a heavy atom effect on the Paternò–Büchi reaction based on the phenomena noted above. The formation of two regioisomers in the Paternò–Büchi reaction is detailed in Scheme 2.

Among these processes, there are four factors that determine the regioselectivity [7]. (i) Initial *O*-attacked site selection at the double bond by ^3^benzophenones*, *k*_2a_+ *k*_2a’_ versus *k*_3a_+ *k*_3a’_, (ii) equilibrium constants, *K***_2_** (= [C5-^3^DR2]/[C5-^3^DR1]) versus *K***_3_** (= [C6-^3^DR2]/[C6-^3^DR1]): This factor only operates when the conformational change is faster than the ISC process, (iii) the relative rate constants of the ISC processes in the triplet 1,4-diradicals, *k*_2I_/*k*_2I’_ versus *k*_3I_/*k*_3I’_ and (iv) the relative rate constants of the bond-forming and bond-breaking step from the singlet 1,4-diradical C5-^1^DR1 and C6-^1^DR1, *k*_2b_/*k*_2c_ versus *k*_3b_/*k*_3c_.

According to the Curtin–Hammett principle [20], the ratios of the productive conformers of singlet diradicals C5-^1^DR1 and C6-^1^DR1 are determined not only by the populations of C5-^3^DR1 and C6-^3^DR1 but also by the relative rate constants of ISC processes, the *k*_2I_/*k*_2I’_ and *k*_3I_/*k*_3I’_. The former is determined by the equilibrium constants, *K***_2_** and *K***_3_**, whilst the latter processes (*k*_2I_, *k*_2I’_, *k*_3I_ and *k*_3I’_) would be accelerated by heavy atoms. Thus, the equilibrium between the productive conformers and the unproductive conformers, of the triplet 1,4-diradicals, would be achieved at a higher temperature for the system with heavy atoms than that without heavy atoms. Due to the energy barriers between the two stable conformers [9], the equilibrium is more favorable for the formation of oxetanes **3** rather than oxetanes **2** at a higher temperature. This would lead to a higher inversion temperature and a higher ratio of **2**/**3**. In addition, the ISC process from singlet excited state to triplet excited state is very fast, 10^11^ s^−1^ for benzophenones, and not affected by heavy atoms, but the ISC process of triplet benzophenones to singlet ground state would be accelerated, reducing lifetime of triplet benzophenones. Finally, triplet benzophenones with a short lifetime would give rise to a less efficient Paternò–Büchi reaction.

## Experimental

### Materials

1,3-Dimethylthymine (DMT) and 1,3-dimethyluracil (DMU) were was prepared from thymine and uracil, respectively. 4,4’-Dimethoxybenzophenone, 4,4’-dichlorobenzophenone, 4,4’-dibromobenzophenone and 4,4’-dicyanobenzophenone were prepared. Benzophenone, 4,4’-difluorobenzophenone, acetonitrile-*d*_3_ and other materials were obtained from commercial suppliers and used as received without further purification. ^1^H and ^13^C NMR spectra were measured with a Bruker AV 300 spectrometer operating at 300 MHz and 75 MHz, respectively.

The Paternò–Büchi reaction of DMT/DMU with benzophenones generates two regioisomers **2** and **3**. The oxetanes were numbered by using subscript “1” for DMT and “2” for DMU, e.g., oxetanes from DMT-**1a** system are denoted as **2a_1_** and **3a_1_**. For oxetanes mentioned in this work, most were reported in our previous papers [9–10] except for the following oxetanes. **3a_2_** has not been detected by ^1^H NMR. **3d_1_**, **3d_2_**, **3e_1_**, **2e_2_** and **3e_2_** could be detected by ^1^H NMR, but could not be isolated because of their poor stability towards acid and silica gel. **2e_1_** was isolated and the characterization data of **2e_1_** was as follows:

**8,8-Bis-(4-bromo-phenyl)-2,4,6-trimethyl-7-oxa-2,4-diaza-bicyclo[4.2.0]octane-3,5-dione (2e_1_)**. ^1^H NMR (300 MHz, CDCl_3_) δ = 1.73 (s, 3H, CH_3_), 2.91 (s, 3H, NCH_3_), 3.11 (s, 3H, NCH_3_), 4.50 (s, 1H, NCH), 7.13–5.54 (m, 8H, H_Ar_) ppm; ^13^C NMR (75 MHz, CDCl_3_) δ = 24.1, 27.6, 35.9, 66.8, 91.0, 122.5, 122.8, 126.8, 127.4, 131.8, 132.1, 137.6, 142.8, 151.6, 169.6 ppm; IR (KBr) 3435 (*s*), 2930 (*w*), 1704 (*m*), 1673 (*s*), 1484 (*s*), 1282 (*s*), 1068 (*s*), 1008 (*m*), 818 (*m*), 746 (*m*) cm^−1^; TOFMS (CI) *m/z* calcd for (M+H)^+^ C_20_H_18_N_2_O_3_Br_2_: 494.9742, found 494.9731.

### Photoproduct assay

The Paternò–Büchi reactions of DMT/DMU with benzophenones were performed in acetonitrile-*d*_3_. A solution of the reactants was placed in a Pyrex NMR tube (transmitted light > 290 nm), purged with high purity N_2_ for 10 min and then irradiated with a 125 W high-pressure Hg lamp at 10 °C. The sample tubes were placed on a merry-go-round equipment moving around the Hg lamp. Photoproducts **2** and **3** have no significant absorption for light at above 290 nm. Hence, a secondary photolysis of the oxetane products (**2** or **3**) should not occur unless there is prolonged irradiation. Compositions in photoreaction mixture were quantified by ^1^H NMR spectroscopy (300 MHz) directly on the crude product mixture, using the sum of 5-methyl (5-H) and 6-H signals as internal standards. The yields and the ratios of the two regioisomeric oxetanes were obtained from the peak areas of 5-methyl and 6-H for DMT system and those of 5-H and 6-H for DMU system in the ^1^H NMR spectra. The experimental error was within 5%.
